# Biocompatibility of Different Nerve Tubes

**DOI:** 10.3390/ma2041480

**Published:** 2009-09-30

**Authors:** Felix Stang, Gerburg Keilhoff, Hisham Fansa

**Affiliations:** 1 Department of Plastic, Reconstructive and Hand Surgery, University of Luebeck, 23538 Luebeck, Germany; 2 Institute of Biochemistry and Cell Biology, University of Magdeburg, 39120 Magdeburg, Germany; E-Mail: gerburg.keilhoff@med.ovgu.de; 3 Department of Plastic, Reconstructive and Aesthetic Surgery, Hand Surgery, Klinikum Bielefeld-Mitte, 33604 Bielefeld, Germany; E-Mail: hisham.fansa@klinikumbielefeld.de

**Keywords:** nerve tube, nerve repair, biocompatibility, artificial nerve, peripheral nerve

## Abstract

Bridging nerve gaps with suitable grafts is a major clinical problem. The autologous nerve graft is considered to be the gold standard, providing the best functional results; however, donor site morbidity is still a major disadvantage. Various attempts have been made to overcome the problems of autologous nerve grafts with artificial nerve tubes, which are “ready-to-use” in almost every situation. A wide range of materials have been used in animal models but only few have been applied to date clinically, where biocompatibility is an inevitable prerequisite. This review gives an idea about artificial nerve tubes with special focus on their biocompatibility in animals and humans.

## 1. Introduction

Peripheral nerve repair is still a challenge in reconstructive surgery. When primary, tension free nerve coaptation is not possible, replacement of a missing nerve segment with an (usually non-vascularized) autologous nerve graft is the gold standard.

The use of cutaneous nerves as grafts, which until today was considered to be the gold standard, was already suggested 1915 by Foerster [1]. The sural nerve is the most commonly used donor nerve [1]. Other suitable donor sites are the lateral and medial antebrachial cutaneous or posterior interosseus nerves. In contrast to a vessel graft, a nerve graft is not a “plug’n play”-tool. The graft must not only cover the defect between the proximal and distal nerve stump, it must also provide the optimal conditions and environment for axonal regeneration, such as Schwann cells, revascularization and endoneurial morphology.

**Figure 1:**
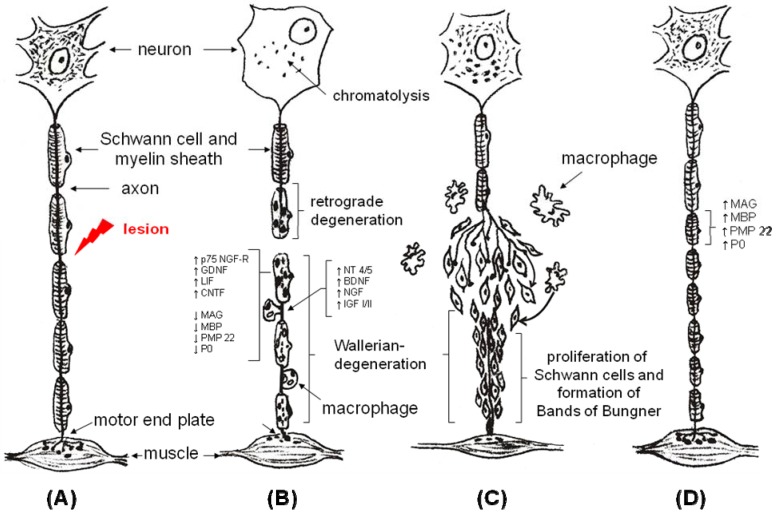
Wallerian degeneration and axonal regeneration.

After a lesion of a peripheral nerve (A) retrograde and anterograde Wallerian degeneration takes place (B). This includes a “switching” of the Schwann cells from a “non-myelinating” modus into a “myelinating” modus with complex changes in the genetic program of Schwann cells including downregulation of factors like myelin associated glycoprotein (MAG), myelin basic protein (MBP), peripheral myelin protein 22 (PMP 22) and protein zero (P0); simultaneously there is an upregulation of p75-nerve growth factor-receptor (p75 NGF-R), glial derived neurotrophic factor (GDNF), leukemia inhibiting factor (LIF) and ciliary neurotrophic factor (CNTF). Schwann cells start proliferating and forming cell columns (band of Bungner), In the mean time there is an invasion of macrophages, clearing the cell debris (C). The proximal axon stumps create a couple of growth cones, which are able to find their way among the pathfinders. Finally, Schwann cells form their myelin sheath around the regenerated axon and change their genetic program again (D). Modified from [2].

The condition for a successful nerve grafting is how well it copes with the challenge of providing optimal conditions for regenerating axons, namely a 100% survival of all transplanted nerve structures after Wallerian degeneration (Figure 1), including the susceptible structures of the endoneurium (basal lamina and contact molecules) and Schwann cells. Nevertheless, success is limited by several factors such as: (1) size of the defect, (2) quality of the wound bed and surrounding soft tissue, (3) quality of vascularization of the donor nerve, (4) alteration of the fascicular architecture in the graft and (5) limited availability of donor nerves.

Furthermore, nerve grafting implicates two suture lines at the proximal and distal coaptation side, which can lead to “neuroma-in-continuity”-formation, due to increased fibrosis and scar tissue formation especially at the distal coaptation side, which “close the door” to the distal nerve stump. Unsolved problems are also apoptosis of motoneurons, especially in nerve lesions close to the neuron and a limited time frame in which muscles can be reinnervated by successful reconnection of the motor end plate to regenerated axons.

Although sensory nerves somehow inhibit regeneration of motor nerves [3,4]—which is a rather irrelevant finding for clinical use, no tubular or other type of conduit has proved to be superior in comparison with the autologous nerve graft, at least not for reconstruction of substantial human motor nerves, such as the ulnar or median nerve trunk.

The ideal nerve reconstruction technique should be one that: (1) permits immediate nerve reconstruction at time of injury, (2) allows a tension free nerve suture, (3) does not require sacrificing a donor nerve, (4) does not add additional intraoperative time, (5) does not place foreign material permanently into the body, (6) does not require additional medication (e.g. immunosuppressive therapy in the case of allografts), (7) does not create a potential nerve entrapment site and (8) that permits concepts of modern neurobiology to enhance nerve regeneration (e.g., specific drug release of growth factors).

## 2. The Tube Concept

To overcome the limitations of autografts, intensive research is being done investigating artificial nerve tubes [5]. Nerve regeneration through an artificial nerve tube can ideally be described with five different stages [6]: (1) during the first day, the tube is filled with a fluid possibly containing neurotrophic factors; (2) from days 2 to 6, an acellular fibrin matrix forms in order to bridge the proximal and distal nerve stump; (3) this is followed between days 7 to 14 by an infiltration of Schwann cells, migrating from the distal and proximal stump, and endothelial cells; (4) then, between days 15 and 21, axons begin to grow from the proximal stump, increasing in size and becoming myelinated; (5) finally, regenerating axons enter the distal nerve stump after an appropriate time frame, which depends on length of the gap.

**Figure 2:**
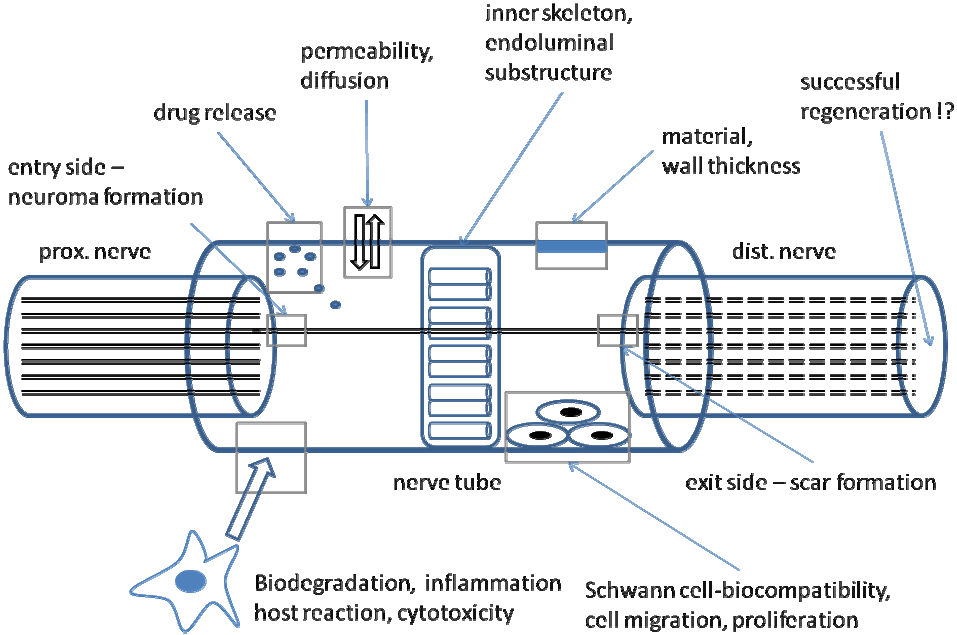
Different aspects of an artificial nerve tube.

A major problem of all hollow tubes is misdirection: since transected axons produce axon sprouts proceeding in a distal direction, a neuroma is always created, consisting of minifascicles proceeding in an irregular way, proliferating Schwann cells, fibroblasts and capillaries. If there is a directional factor of any kind, e.g., an artificial nerve tube which usually provides no endoneurial structure, the neuroma proceeds in the desired direction. Schröder and Seiffert called this phenomenon “neuromateous neurotization” [7]. In a consequence, only few dispersed axons are able to enter the right fascicle and endoneurial tube in the distal nerve stump once they have reached the end of the tube. These probably misdirected axons may lead to inappropriate target reinnervation or polyinnervation of different targets by dispersion of axonal branches originating from the same motoneuron [8]. Clinically this results in either no reinnervation or co-contractures. Therefore, first demand for artificial nerve tubes is a physical substructure or inner skeleton, offering endoneurial-like tubes comparable to an autograft [9] (Figure 2).

The first aspect is the kind of material, including wall thickness and structural integrity in order to prevent collapse. Wall permeability enables diffusion and transport of growth factors through the wall and might enable a defined release of drugs like certain growth factors. The tube should offer some kind of endoluminal substructure in order to prevent neuroma formation at the entry side or scar formation at the exit side. The material should be biodegradable without any significant cytotoxic reactions and it should be a suitable matrix for regenerative cells like Schwann cells, offering contact molecules for cell migration and cell adhesion.

## 3. Biodegradability

After successful nerve regeneration, the artificial nerve tube becomes useless or may even have adverse effects on the regenerated nerve fibres. Therefore, nerve tubes should preferably be “biodegradable” with rapid decomposition leaving no trace after serving their function. The speed of decomposition of nerve tubes should match with axonal growth rates and with respect to the gap length. The tubes material and its degradation products should be non-mutagenic, non-toxic, non-carcinogenic and non-immunogenic. Besides this, they should not cause any local or systemic irritation or allergic reaction. For polymer degradation (see below), the prevailing mechanism is a simple chemical random hydrolysis of the hydrolytically unstable ester bonds backbone. This can be described with two stages: (1) water out of the intercellular space penetrates the bulk of the device, preferentially attacking the chemical bonds in the amorphous phase and converting long polymer chains into shorter water-soluble fragments. This goes along with some swelling of the device and a reduction of molecular weight but without a loss in physical properties, since the device matrix is still held together by the crystalline regions. (2) an enzyme-catalysed hydrolysis and metabolization of the fragments by macrophages occurs, resulting in a rapid loss of polymer mass. Concomitantly, the pH inside pores begins to be controlled by degradation products, which typically have some acid-base functionality. In the case of polyglactin, pH drops down to a level of less than 4.7 and osmolarity approximately doubles to the value of 600 mosml/L. Impairment of nerve regeneration *in vivo* by such changes cannot be excluded [10]. This type of degradation is called bulk erosion and as a matter of principal, all synthetic polymer materials degrade by bulk erosion [11].

Modern neurobiology provides detailed insights of the regenerating process and gives plenty of ideas how to improve nerve regeneration. Simply, two different approaches can be identified. First, the implantation of viable Schwann cells [12,13,14,15,16], which could be cultivated out from human neuromas [17]. These Schwann cells can provide several trophic and topic factors within the correct time frame. New articles even describe the use of genetically engineered Schwann cells to improve nerve regeneration [18,19,20]. The second approach is the selective use of neurotrophic factors such as FGF, NGF, CNTF or BDNF which are included in “drug delivery systems” within the nerve tube [21,22,23,24,25].

## 4. Revascularization and Physical Properties

Revascularization or angioneogenesis with an adequate supply of nutritional factors is another important requirement. Usually, biological nerve grafts made from acellular muscle or collagen are usually revascularized within the first 4-5 days after implantation by longitudinal ingrowth of vessels from the distal and proximal nerve stump and sprouting of collateral capillaries [26,27]. Before that, nutrition depends on diffusion through the tubes’ wall. Permeable scaffolds should allow the influx of externally generated wound healing factors and the outward diffusion of waste products. Of course, neurotrophic factors produced by Schwann cells should stay inside the lumen. However, impermeable conduits can positively affect nerve regeneration by insulating the area of regeneration, preventing the ingrowth of scar tissue formation and by keeping internally generated growth factors inside [28]. Semi-permeable tube walls may also facilitate the formation of a supportive fibrin cable (acting as some kind of “longitudinal guiding structure”) by allowing inward diffusion of extraneural wound-healing factors [29]. Improved nerve regeneration and successful reinnervation have been shown to occur mainly in semi-permeable nerve conduits [30,31,32,33]. Only little data are available concerning the revascularization of polymer-tubes.

Nerve conduits with thick walls are more rigid with impaired handling and difficult suturing under the microscope. Furthermore, they possess poor *in vivo* tissue compatibility due to rigidity and therefore more likely provoke local inflammation reactions. It is reported that regenerated axons were significantly longer in tubes with an average wall thickness of 0.81 mm when compared to those in tubes with thicker wall of 1.1 mm, 1.28 mm and 1.44 mm [34]. Thin walled conduits are also associated with less neuroma formation, which was attributed to the greater elasticity of thin walls [35]. Up to date, no nerve tube with a wall thickness of less than 100 µm has been reported for peripheral nerve reconstruction. Very thin walls can lead to collapsing of the tube *in vivo*, for example, amorphous poly(D,L-lactide-ε-caprolactone) tubes with an average wall thickness of 170 µm collapsed 26 weeks postoperatively [36]. Meek *et al*. reported of a 40% collapse of all nerve tubes made of electrospun PCL/PLGA fibres with a wall thickness of 150 µm by four months after surgery [37].

Beyond this, several other important properties of artificial nerve tubes have to be kept in mind. They should be easily fabricated with the desired dimension and topography, comfortable in handling and implantation, being of low cost and of course sterilizable.

Different materials, some of them already with a wide application in human medicine, have been suggested to be an appropriate substitution of autologous graft (Table 1). On the one side there are biological nerve grafts, including arteries, veins [38,39,40] or muscles [40,41,42,43] as well as modified biological tissues such as fibrin [44], various types of collagen [9,45,46] or spider silk fibres [47].

**Table 1:** Overview of different biological and synthetic nerve grafts.

**Biological Nerve Grafts**	Animal model (A) / Clinical trial (C)
veins	A, C
acellular muscles	A, C
Collagen type I/III	A, C (NeuraGen®, Neuramatrix®)
spider silk fibers	A
acellular nerves	A, C (AxoGen®)
**Synthetic Guidance Channels**	Animal model (A) / Clinical trial (C)
poly(D,L-lactide-caprolactone)	A, C (Neurolac®)
polyglycolic acid	A, C (Neurotube®)
poly (2-hydroxyethylmethacrylate-co-methyl methacrylate)	A
poly-3-hydroxybutyrate	A
silicone	A,C
glass fibers	A

On the other side we have a wide range of synthetic polymer guidance channels such as polyglycolic acid (PGA, Neurotube^®^) [48,49,50,51], poly(D,L-lactide-ε-caprolactone) (PLA, Neurolac^®^) [52,53], poly(2-hydroxyethylmethacrylate-*co*-methyl methacrylate) (PHEMA-MMA) [54,55] or poly-3-hydroxybutyrate (PHB) [56]. Even silicone tubes [57,58,59,60,61,62,63] or glass fibres [64,65,66] have been reported. Some of these materials are already used in several clinical human trials (Table 1). Meek and Coert previously gave an overview of materials approved by the US Food and Drug Administration [67]. A search of the Medline database reveals that new materials or techniques for optimizing peripheral nerve reconstruction are constantly being described. This review gives an overview of different materials used for artificial nerve guides and currently described in literature, with special focus on their biocompatibility.

## 5. Silicone Grafts

A silicone polymer tube consists of high molecular weight compounds made of silicon, oxygen and hydrogen. Its characteristics including elasticity can be modified by specific selection of organic side-chains and the ratio of side-chain lengths to crosslinks. Silicone is hydrophobic, and considered to be well accepted physiologically. It is impermeable and not biodegradable, and therefore broadly used, for example, in plastic surgery (e.g., breast implants). But clinical experience using silicone nerve tubes is very limited. Merle *et al*. were the first using silicone guides clinically [68] and reported successful nerve regeneration. However, after two years, nerve compression syndromes occurred at the implantation site so that a second operation was required to remove the silicone tube. Other authors reported similar experiences [69,70,71]; nevertheless silicone is still used in animal models to investigate peripheral nerve regeneration [57,58,62,72].

## 6. Acellular Muscle Grafts

**Figure 3:**
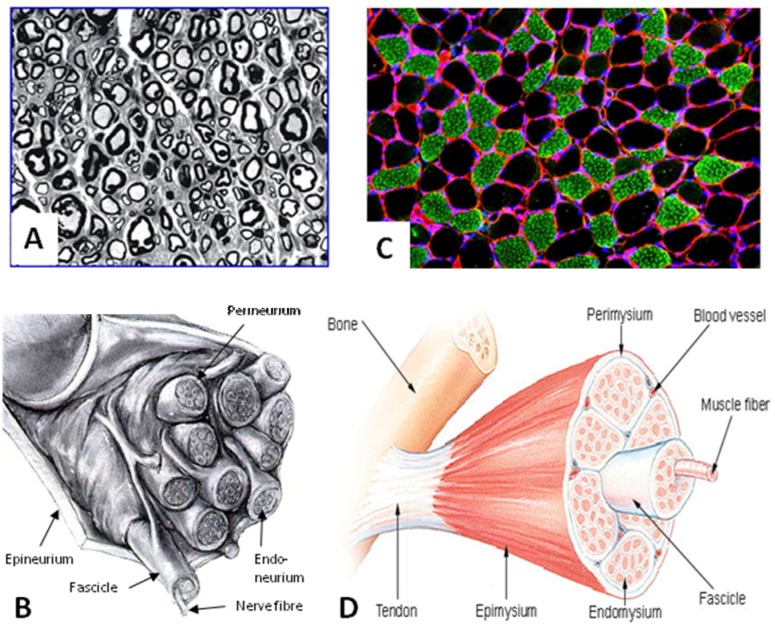
Macro- and microanatomy of a nerve and muscle.

After recognizing the importance of an inner skeleton and basal lamina to promote axonal regeneration, research focused on acellular muscles as nerve tubes. Internally a coaxially aligned acellular muscle consists of a bundle of ideally empty cylinders which apparently offer no or little resistance to ingrowing Schwann cells and regenerating axons (Figure 3 C). Furthermore, the tubes’ walls contain contact molecules such as laminin, fibronectin or collagen type IV acting as axonal pathfinders. Devitalized muscle grafts provide an effective matrix for implanted Schwann cells to adhere to, and the remaining muscle basal lamina tubes offer spaces for the regenerating axons to grow through [73]. Within such basal lamina tubes, cell columns (Bungner bands) are formed as a pathway through which regenerating axons grow to reach their target organ. From a microscopic perspective, acellular muscle grafts mimic endoneurial tubes comparable to the autograft (Figure 3). *In vivo*, acellular allogenic muscle grafts show only a moderate host reaction in terms of expression of major histocompatibility complex I and II and invasion of CD4/CD8 positive cells and macrophages [74]. Revascularization takes place five days after implantation [27].

**Figure 4:**
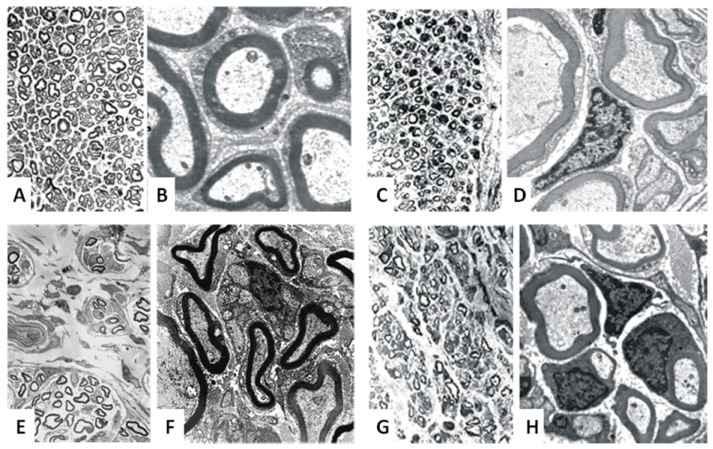
Histological evaluations of regeneration results of different nerve tubes.

Initially the method of denaturation was to freeze a piece of muscle in liquid nitrogen and then osmotically shock the tissue by immersing it in distilled water. A couple of modifications have been described in literature, including the implantation of Schwann cells into the graft [14,73]. Our own experiments showed the acellular muscle graft to be an good option with promising regeneration results [40,75,76], even in gaps of up to 5 cm [43] (Figure 4). Acellular muscle grafts are even a suitable matrix for transdifferentiated mesenchymal stem cells, which can support peripheral nerve regeneration to certain extent [77,78]. Norris and colleagues first described digital nerve reconstruction in humans using a skeletal muscle autograft [41]. In 1991 Pereira and colleagues published a clinical study in humans with encouraging preliminary results [79,80,81], but no further studies followed.

(A) Microscopical cross-section of a peripheral nerve, with plenty of myelinated nerve fibres. (B) Macroscopically, the nerve consists of fascicles, in which the nerve fibres are embedded into the endoneurium. The acellular muscle shows a similar histological structure with endoneurial-like tubes (C). Nerve and muscle show a similar architecture with corresponding substructures (D). Source: B: [82], C: [83], D: [84].

## 7. Vein Grafts

In 1909 Wrede was the first to report about the use of a vein as nerve conduits in a 27-year old patient with a median, ulnar and medial antebrachial cutaneous nerve defect of 7 cm [85]. Several clinical cases followed and seemingly showed effectiveness of this method in the return of sensation using a vein graft in defects shorter than 3 cm. Today, the use of veins for finger nerve reconstruction (e.g., in digital replantation) is accepted within literature [38,86], although it includes a donor site. Veins may collapse due to their thin walls and surrounding scar tissue can cause constriction especially over long distances. In addition, vein valves may function as a total block to regenerating axons. Within our own experiments, acellular muscles grafts proved to be superior to veins in a 2 cm gap [40].

(A) and (B) show a nerve autograft with homogenous spread nerve fibers with regular myelin sheaths. (C) and (D) show an acellular muscle graft, seeded with Schwann cells. The myelin sheaths appear to be more irregular. A collagen graft shows only particularly associated nerve fibres with more irregular myelin sheaths. Acellular muscle grafts seeded with transdiffentiated mesenchymal stem cells support nerve regeneration to a certain extent - with myelin sheaths comparable to the acellular muscle graft (G, H).

## 8. Fibrin Grafts

Fibrin (also called Factor Ia) is a fibrillar protein involved in blood clotting. Polymerized into a "mesh", it creates a hemostatic clot (in conjunction with platelets) over a wound site. Fibrin is made from fibrinogen, a soluble plasma glycoprotein. Processes in the coagulation cascade activate the zymogen prothrombin to the serine protease thrombin which is responsible for converting fibrinogen into fibrin. Fibrin is then cross linked by factor XIII to form a clot. Fibrin glue is widely used in surgical practice, helping surgeons to adept tissues and to obtain hemostasis [87]. Fibrin and its non-toxic degradation products induce angiogenesis and promote cell attachment and proliferation. Some reports even describe good results in nerve coaptation with fibrin glue [88,89].

Kalbermatten and colleagues have recently described a novel nerve tube made of fibrin glue [44]. After one week *in vivo* they found an intact structure with obvious porosity and no signs of hematoma or infection. But already after two weeks *in vivo*, fibrin conduits showed a partial resorption, with an approximate diameter-reduction of 20%. This indicates that a “single”-fibrin matrix might not be stable enough to serve as a scaffold for longer distances. Laidmae demonstrated low toxicity of salmon fibrin implanted intraperitoneal into mammalians with no deleterious effects on coagulation profiles and no cross reactivity with host fibrinogen or thrombin [90]. Using a three dimensional salmon fibrin matrix, Ju *et al* showed an enhanced neurite growth from mammalian neurons, likewise with more resistance to proteolysis than mammalian fibrins [91]. A combination of fibrin matrix and Schwann cells within an artificial nerve conduit (poly-3-hydroxybutyrate) can enhance peripheral nerve regeneration [56].

## 9. Collagen Grafts

Collagen is the main protein of connective tissue in animals and humans. Tough bundles of collagen called collagen fibers are major component of extracellular matrix that supports most tissues and provides structure. Collagen has great tensile strength. There are more than 28 types of collagen described in literature. However, more than 90% of the collagen in the body is of type I, II, III, and IV. Type I collagen is the predominant collagen in the intact peripheral nerve and constitutes together with collagen type III 49% of total protein in nerves [92]. Since collagen is a natural material, it shows excellent biocompatibility, insignificant immunogenicity, and high bio-absorbability. Thumann and colleagues proved that collagen is non-toxic by morphology, viability and differentiation analysis. After 24 weeks of subconjunctival implantation, collagen membranes were non-toxic and did not elicit any rejection or inflammatory response. Collagen was successfully absorbed in 17weeks [93].

Microgeometry and permeability (up to 100,000 Dalton) allows diffusion through the collagen wall [94], whereupon a molecular weight cut-off of approximately 50,000 Dalton has been found suitable to allow diffusional transport of nutrients and other molecules while preventing cells from entering the conduit [6]. The adhesive properties of collagen allows settlement and proliferation of Schwann cells and angioneogenesis [26], which is clearly superior to silicone tubes [95].

Degradation of collagen tubes has been reported to occur after 1 to 36 months, depending on the different fabrication methods. By cross-linking with polyepoxides or photochemical cross-linking, degradation rate can be modified [96,97,98]. If collagen is partially hydrolyzed under mild conditions, the three collagen strands separate into globular, random coils, producing gelatin, which has in contrast to collagen a relatively lower antigenicity.

Already more than 25 years ago, Mundy and colleagues found chemotactic effects of collagen and collagen-derived fragments for tumor cells [99] and recently it was shown that type I collagen receptor signaling promotes the growth of human prostate cancer cells within the bone [100]. Although adverse side effects of collagen nerve tubes on tumor formation cannot be ruled out, we are unaware of a single case of tumor formation after collagen implantation.

Due to its nature, collagen type I is often used in medical implants such as artificial skin grafts (e.g., Dermal Regeneration Template, Integra®), wound dressings and nerve conduits due to its low immunogenicity, *in vivo*-biocompatibility and its absence of hosts body reaction [101]. In a rat sciatic nerve model, our own results using collagen type I/III nerve tube showed only moderate inflammatory host response in terms of invasion of ED1 positive macrophages and collagen proofed to be a suitable matrix for Schwann cells [26].

Bozkurt developed a novel, 3-dimensional (3D), highly oriented and cross-linked porcine collagen scaffold to promote directed axonal growth of dorsal root ganglia *in vitro*. After 21 days he showed S100-positive Schwann cells (SCs) migrating into the scaffold and aligning within the guidance channels in a columnar fashion, resembling "Bands of Bungner." Neurofilament-positive axons (mean length +/- SD 756 µm +/- 318 µm, maximum 1,496 µm) from dorsal root ganglia neurons entered the scaffold where the growth within the guidance channels was closely associated with the oriented SCs [102].

Due to these abilities, collagen nerve tubes have been used extensively in the peripheral nervous system experimentally for nerve repair [9,26,45,72,103,104,105] and currently two different collagen tubes (*NeuraGen^®^* from Integra Neuroscience, www.integra-ls.com and *Neuromatrix/Neuroflex^®^* from Collagen Matrix Inc., www.collagenmatrix.com) are approved by the FDA for clinical use [67].

Archibald introduced a collagen tube produced by Integra Lifescience (later the *NeuraGen^®^* tube) in a monkey median nerve model. Bridging four median nerve gaps of 5 mm with a collagen tube he showed a final level of physiological recovery compared to the autograft and direct nerve suture [104]. Today, *NeuraGen^®^* tubes are available in different sizes with lengths of 2 or 3 cm and inner diameters of 1.5 to 7 mm. In 2005 Taras reported positive clinical impressions with the repair of 73 peripheral nerves utilizing the *NeuraGen^®^* tube. Repaired nerves include median, ulnar, radial, posterior interosseous, common digital, and the superficial radial sensory nerve - unfortunately no single outcome value is presented, which makes this report debatable [106]. Currently three studies with *NeuraGen^®^* tubes used in patients are published. In 2006 Ashley *et al*. reported about five patients with obstetrical brachial plexus lesion [107]. Four of them showed a good regeneration result after two years. Lohmeyer published results of 15 nerve repairs in the hands of 14 patients. The longest gap was 18 mm and 12-month measurements could be obtained from 12 patients. Four of them showed excellent results (S4), five patients achieved good sensibility, one patient poor and two patients no recovery at all [108]. Farole and Jamal recently reported of eight female patients who received a total of nine *NeuraGen^®^* cuffs for repair of six lingual and three inferior alveolar nerves. Four cases were found to have good improvement, four some improvement and onr had no improvement (classification of Progel). None of the cases had worsening of symptoms [109]. Further studies are going on with a multicenter study within Germany and will be published soon. Ahead, we observed several patients in whom the collagen implant had to be removed, but more likely due to super-infection, poor tissue coverage or persistent neuroma pain. Although approved since 2001, no clinical study has been published yet using the new *NeuroMatrix/Neuroflex^®^* system.

## 10. Glass Fibre Grafts

Controlled release glasses are a new class of materials. They are inorganic polymers, normally based on phosphates of sodium and calcium, which have been converted into a glassy form by melting the constituents at about 1,000 ºC. They are biodegradable and dissolve in water completely, leaving no solid residue. The composition of the glass can be changed by using elements other than sodium and calcium. In applications of controlled release glasses, no ill effects or reactions were observed. In an extensive experiment with five sheep, the facial nerve was repaired by entubulation with biodegradable glass tubes. After 10 months animals were assessed and compared with a similar sized group of sheep. While there was a reduction in the peak velocity of conduction in the repaired nerves and in the range of conduction velocities, the minimum conduction velocity was within normal limits. At the time of assessment all of the glass tubes were found to have dissolved completely. Nerve regeneration had taken place to a degree at least as effective as seen in nerves of a similar size repaired by conventional means [66]. Since then, no further reports about glass-nerve tubes could be detected, but in 2007 the same authors used a flexible biodegradable glass fibre wrap for repair of divided median nerves of sheep and compared it to microsurgical epineurial repair. They observed no more fibrosis in the glass fibre wrap groups than in the epineurial repair group and the glass fibre wrap was not present macroscopically at seven months [65]. Bunting and colleagues showed successful Schwann cell growth on glass fibres. By placing glass fibres into a silicone graft and bridging a 0.5 cm interstump gap in the sciatic nerves of adult rats, they demonstrated axonal regrowth at 4 weeks, which was indistinguishable from that which occurs across an autograft [64]. Unfortunately, a 5 mm gap-model is not suitable to proof grafts’ regeneration potential, since virtually every graft placed into a 5 mm gap of a rat leads to good regeneration results.

## 11. Polyglycolic Acid Grafts

Polyglycolide or polyglycolic acid (PGA) is the simplest biodegradable linear aliphatic polyester. In 1962 it was used in the first totally synthetic absorbable suture (Dexon^®^). Glycolide monomers are synthesized starting from glycolic acid by means of polycondensation or ring-opening polymerization, which yields high-molecular-weight materials, with approximately 1–3% residual monomer present. PGA is highly crystalline (45–55%), thus insoluble in water and most organic solvents, with a glass transition temperature about 36 °C and a high melting point (224–228 °C) [11,110] . Fibers of PGA exhibit high strength and are particularly stiff. To reduce stiffness, PGA has been copolymerized with other monomers. Currently PGA and its copolymers (poly(lactic-co-glycolic acid) with lactic acid, poly(glycolide-co-caprolactone) with ε-caprolactone, and poly(glycolide-co-trimethylene carbonate) with trimethylene carbonate) are widely used as a material for tissue engineering or controlled drug delivery [11].

Exposed to physiological conditions, PGA is degraded by random hydrolysis and apparently it is also broken down by certain enzymes, especially those with esterase activity. The degradation product, glycolic acid, is non toxic and it can enter the tricarboxylic acid cycle after which it is excreted as water and carbon dioxide. A part of the glycolic acid is also excreted by urine [11]. During the degradation process, a transient pH-decline is observed, therefore the amount of implanted polymer material should be limited [10,111]. After two weeks, sutures made of PGA have lost about 50% of their strength and 100% after four weeks. The polymer is completely resorbed by the organism in a time frame of six to eight months [112,113]. By, for example irradiation of the compounds to partially break covalent linkages within the polymer, the hydrolytic process can be influenced.

In general, PGA and PLA biomaterials have demonstrated satisfactory biocompatibility and absence of significant toxicity, although some reduction in cell proliferation has been reported. PLA-PGA copolymers have also been frequently used in bone repair applications and have been found to be biocompatible, non-toxic and non-inflammatory. PGA has also been considered to be immunologically inert, following cytological analysis of materials aspirated from malleolar fracture repair effusions developed around PGA implants, although inflammatory monocytes were observed [114].

In 1983 Rosen and colleagues already used a thin walled PGA-tube for repair of a transected rat peroneal nerve [115], with minimal host response after 10 weeks. In recent times, Nakamura and colleagues suggested PGA for peripheral nerve reconstruction after successful implantation of PGA-collagen tubes in 15 mm gaps of 24 dog peroneal nerves [51]. Waitayawinyu compared PGA tubes and type I collagen nerve tubes with the gold standard of autogenous nerve grafting. Bridging a 10-mm segment of 45 rat sciatic nerves, axonal sprouting was significantly less organized and less dense with the PGA conduits when compared to nerve reconstruction with the type I collagen conduits and nerve grafts [116].

For peripheral nerve reconstruction in humans, in 1990 Mackinnon and Dellon reported about 15 patients in a prospective study of secondary nerve reconstruction between 5 to 30 mm with a PGA mesh that had been rolled and heat welded to form a tube *(later Neurotube^®^, Synovis, www.synovismicro.com).* Only two patients showed poor recovery, whereas thirteen patients achieved good or excellent results [117]. They concluded PGA to be as equivalent to autologous nerve graft and in 1992 they reconstructed the inferior alveolar nerve of a patient with nerve avulsion during extraction of the third molar. At six months, neurosensory testing demonstrated sensory recovery in the lower lip, which by one year was approximating normal sensation in the lip [118]. Several reports about successful repair of median plantar nerve [119], facial nerve [120], spinal accessory nerve [121] and median nerve followed [122]. Navissano and colleagues published a study of seven posttraumatic lesions of terminal branches of the facial nerve repaired with the *Neurotube^®^.* Gap length varied between 1 and 3 cm; 7-12 months postoperatively muscle function was very good in one case, good in four, and fair in two (71% positive results). No intolerance or discomfort was reported or observed [120]. The largest and only prospective and randomized multicenter study is described by Weber and colleagues [48], randomizing 136 nerve transections in the hand of 98 patients into two groups (standard repair with autologous nerve graft or direct end-to-end-repair vs. PGA-tube). Excellent results were obtained in 44% of the PGA group, good results in 30% and poor results in 26%. The overall results between the two groups for repairs up to 3 cm did not show significant differences, but when sensory recovery was examined with regard to length of nerve gap, nerves with gaps of 4 mm or less had better sensation when repaired with a conduit (mean moving two-point discrimination 3.7 ± 1.4 mm for PGA-tube repair vs. 6.1 ± 3.3 mm for end-to-end repair). Inada reported of successful motor nerve recovery in two patients with a transected frontal branch of the facial nerve, and using a PGA tube filled with collagen type I/III. The same tube was successfully used in two patients with neuroma pain after transections of a proper digital nerve and superficial peroneal nerve, in each case with recovery of the sensory nerve function [49,50]. Recently, Rosson published a retrospective chart review of six identified patients with bioabsorbable nerve conduit repair of short-gap motor nerve injuries over a 7-year period - all patients had some return of motor and sensory function [123].

## 12. Poly(D,L-lactide-ε-caprolactone) Grafts

Polylactide is the cyclic dimer of lactic acid which exists as a D- and L- optical active isomer. Poly(L-lactide) is the naturally occurring isomer, paracrystalline with high tensile strength and low elongation. Poly(L-lactide) is about 37% crystalline, with a melting point of 174–184 °C and a glass-transition temperature of 57 °C. The degradation time of poly-L-lactide is very slow, requiring more than two years to be completely absorbed [110].

Poly(D,L-lactide) is a synthetic composition of D-lactide and L-lactide. It is an amorphous polymer exhibiting random distribution of both isomeric forms of lactic acid, and accordingly is unable to arrange into an organized crystalline structure. This material is more amorphous and flexible, with lower tensile strength, higher elongation, and a much more rapid degradation time.

Poly-ε-caprolactone is a semicrystalline polymer with a melting point of 59–64 °C and a glass-transition temperature of −60 °C. It is biocompatible and used as a biodegradable suture. Since the homopolymer has a degradation time of approximately 2 years, copolymerization with poly(D,L-lactide) modifies crystallinity pliability and melting point in order to accelerate the rate of bioabsorption. A block copolymer of ε-caprolactone with glycolide, offering reduced stiffness compared with pure PGA, is being sold as a monofilament suture (Monocryl) by Ethicon, Inc. (Somerville, NJ).

Already 25 years ago, Seckel used PLA-nerve tubes with successful regeneration in nerve gaps less than 10 mm [124] and so did Evans and colleagues [15,52,53]. Recently Lu and colleagues evaluated a multi-layer microbraided PLA-fiber-reinforced conduit for peripheral nerve regeneration. Bridging a 10 mm sciatic nerve gap of rats no clinical problems were observed but an acute inflammatory response, characterized by rapid accumulation of lymphocytes and macrophages, decreasing after 4 weeks and becoming chronic [125]. Later, den Dunnen used the copolymer poly(L-lactide-ε-caprolactone) with successful assembling of regenerating axons as normal nerves over a 10 mm gap after ten weeks [126].

For clinical use a tube named *Neurolac^®^ (Polyganics Inc., www.polyganics.com)* made of poly(D,L-ε-caprolactone) is available [67]. Meek *et al*. published the first preliminary results and reported extensively about the *Neurolac^®^* -tube afterwards [127,128]. They observed several complications. First there is an incomplete degradation of the *Neurolac^®^* -tube with small fragments after 24 months and company of multi-nucleated giant cells and macrophages in terms of a foreign body reaction [129,130]. Furthermore, the *Neurolac^®^* -tube is only flexible after putting it into warm saline before implantation. Needle breaking may occur and the stiffness may lead to inflexibility over joints during mobilization. Finally they observed tube collapse [131], swelling of the biomaterial and automutilation of animals after implantation [132].

## 13. Poly(2-hydroxyethylmethacrylate-co-methyl methacrylate) Grafts

Poly(2-hydroxyethyl methacrylate) (pHEMA) is a polymer that functions as a hydrogel by rotating around its central carbon. In air, the non-polar methyl side turns outward, making the material brittle and easy to grind into the correct shape. In water, the polar hydroxyethyl side turns outward and the material becomes flexible and forms a hydrogel. pHEMA is widely used in contact lenses. Dalton and colleagues described the manufacture of pHEMA tubes for use as nerve guidance channels in a liquid-liquid centrifugal casting [55]. They were able to produce tubes with diverse morphologies, wall thicknesses and mechanical properties and suggested them for repair of spinal cord and peripheral nerve. By bridging a 10 mm gap of a rat sciatic nerve with pHEMA-tubes, Belkas and colleagues showed successful nerve regeneration after eight weeks but sub-optimal regeneration in a minority of the channels after 16 weeks [133]. Tubes were largely biocompatible and most of the tubes maintained their structural integrity up to eight weeks, but 29% of them collapsed by 16 weeks. Some of the 16 weeks tubes showed signs of chronic inflammation and calcification of the tubes [134]. By using coil-reinforced hydrogel pHEMA-tubes, enhanced with FGF-1, the achieved regeneration was equivalent to that of autografts after 16 weeks [54]. So far, no clinical study has been published using pHEMA-nerve tubes in humans.

## 14. Poly(3-hydroxybutyrate) Grafts

Polyhydroxybutyrate (PHB) is a polyhydroxyalkanoate, a polymer of the polyesters class. PHB is produced by certain micro-organisms (like *Alcaligenes eutrophus* or *Bacillus megaterium*) apparently in response to conditions of physiological stress.

PHB is a fully biodegradable polyester with optical activity, piezoelectricity, and very good barrier properties [135]. PHB is a partially crystalline material with a high melting temperature and a high degree of crystallinity.

Microbial biosynthesis of PHB starts with the condensation of two molecules of acetyl-CoA to give acetoacetyl-CoA which is subsequently reduced to hydroxybutyryl-CoA. This latter compound is then used as a monomer to polymerize PHB.

The poly-3-hydroxybutyrate form of PHB is probably the most common type of polyhydroxyalkanoate. Advantages of PHB are its biocompatibility, which makes it suitable for medical applications. It shows good oxygen permeability and is insoluble in water and relatively resistant to hydrolytic degradation. Complete degradation is observed after 24-30 months. This differentiates PHB from most other currently available biodegradable plastics, which are either water soluble or moisture sensitive.

Tohill used a PHB-nerve-tube to implant mesenchymal stem cells into a nerve injury site [136]. These stem cells differentiate into Schwann cells and enhance nerve regeneration [137]. Kalbermatten used a fibrin matrix for suspension of regenerative cells into a PHB-tube. He showed significantly better adhesion of Schwann cells and mesenchymal stem cells to PHB in the presence of fibrin, resulting in a increased nerve regeneration compared to empty PHB-tubes [56]. Young and colleagues investigated the potential of PHB-nerve conduits to bridge long nerve gaps up to 4 cm in a rabbit common peroneal nerve injury model. Regeneration was assessed up to 63 days postoperatively, and compared with that achieved using nerve autografts. By 42 days, regenerating axons had bridged nerve gaps of all lengths in groups with nerve autografts and in those with PHB conduits, but the area of immunostained regenerating fibres in the PHB group was greater than that in the nerve autograft group [138]. Until now, no human trial with PHB-nerve tubes has been reported.

## 15. Acellular Nerve Allografts

Normal nerve allografts inevitably involve the need of immunosuppression with all its drawbacks. Nerve autografts provide good results but are of limited availability. Acellular nerve grafts might serve as a good alternative since they offer the perfect microenvironment in terms of endoneurial tubes and basal lamina.

Several Chinese groups have reported on the use of acellular nerve grafts for peripheral nerve repair. Zhong *et al*. tested chemically extracted acellular nerve grafts in 5 cm dog sciatic nerve gaps. Six months postoperatively they showed a similar regeneration when compared to autografts. Fresh nerve allografts failed due to inflammation and scarring [139]. Niu and colleagues observed longitudinal microvessels throughout acellular grafts and autologous grafts 10, 14 and 21 days after operation; no obvious difference in capillary network of grafts was observed between both groups 28 days after operation; and the microvascular architecture of grafts in both groups were similar to that of normal nerves two and three months after operation [140]. Wang and colleagues implanted autologous bone marrow stromal cells into acellular nerve grafts and evaluated nerve regeneration across a 1-cm lesion in the radial nerve in a rhesus monkey model [141]. Connolly demonstrated successful cavernous nerve regeneration using 5 mm acellular nerve grafts. Animals implanted with acellular nerve grafts demonstrated a significant recovery in erectile function when compared with the group that received no repair, both at one and three months. EMG of the acellular nerve grafts demonstrated adequate intracavernosal pressures by three months (87.6% of the normal non-injured nerves). Histologically, the retrieved regenerated nerve grafts demonstrated presence of host cell infiltration within the nerve sheaths. Immunohistochemically, antibodies specific to axons and Schwann cells demonstrated an increase in nerve regeneration across the grafts over time [142]. Further studies showed that Schwann cells, e.g. obtained from a proximal nerve stump neuroma, are able to settle inside an acellular nerve graft [143,144]. Even growth factors like hepatocyte growth factor or nerve growth factor clearly improve the regeneration potential of acellular nerve grafts [24,145]. Our own results did not show an improvement of nerve regeneration using acellular nerve grafts or epineurial grafts, even by implanting Schwann cells—only predegenerated nerve grafts showed results superior in regard to axon count and histologic appearance in comparison to standard grafts and acellular grafts [75,146].

So far, AxoGen Inc. (www.axogeninc.com) offers acellular human nerve allografts *(Avance^TM^ Nerve Graft)* with a length between 15 and 50 mm and a diameter between 1 and 5 mm. In a rat model, Whitlock *et al*. compared autografts with *NeuraGen^®^* nerve tubes and processed rat allografts comparable to *AxoGen's Avance*^®^ human decellularized allograft product [45]. In a 14-mm sciatic nerve gap model, autografts were superior to processed allografts, which were in turn superior to hollow *NeuraGen^®^* tubes at six weeks postoperatively. At 12 weeks, these differences were no longer apparent. In a 28-mm graft model, autografts again performed better than processed allografts at both, six and 22 weeks; regeneration through the *NeuraGen^®^* tube conduit was often insufficient for analysis in this long gap model.

## 16. Conclusions

The restoration of effective axonal regeneration and nerve function following peripheral nerve injury continues to be a considerable clinical challenge. The autologous nerve graft is considered to be the gold standard. It provides an internal structure with basal lamina tubes and Schwann cells that limit dispersion, as well as good mechanical properties for flexibility and strength. Donor site morbidity is relatively low with approximately 90% of the patients being satisfied with the outcome of the donor site [1] and the operation time can be reduced by operating simultaneously. Nevertheless, a major disadvantage remains the limited supply for repair of extended nerve defects. Nerve allografts implicate the usage of immunosuppressants with all their adverse side effects. Since those sides effects are potentially life-threatening, nerve allografts are usually not used for “non-vital” nerve reconstructions.

The underlying molecular mechanisms of nerve regeneration in terms of neurotrophism, contact guidance and neurotropism are more and more realized. All studies using implanted Schwann cells report about improvement of peripheral nerve regeneration in the presence of Schwann cells. Especially for long nerve gaps, we believe Schwann cells inside a tube to be mandatory. However, chronically denervated Schwann cells change their genetic profile and lose their ability to express and produce genes and neurotrophic factors that assist nerve regeneration [147,148,149]. Since animal studies have demonstrated a potential beneficial adjunct of stem and precursor cells (embryonic neural stem cells, adult stem cells, mesenchymal stem cells, multipotent stem cells from adipose or skin tissue) Walsh and Midha provided practical considerations concerning the use of those cells for peripheral nerve regeneration, specially ideal number and method of cell delivery and the extend of transplant cell survival and differentiation to enhance peripheral nerve regeneration [150].

The theory is simple: we need an artificial nerve tube with Schwann cells (or capable stem cells) that mimic the autologous nerve graft. The ideal structure of inner matrix of a tube is still difficult to develop but it seems that an endoneurial-like substructure is suitable. A variety of different synthetic and biological materials have been used to construct nerve conduits and uncountable reports have been published. Generally it is impossible to compare single studies in terms of “meta-analysis”, since every investigator uses different histological, electrophysiological or functional evaluation methods for peripheral nerve regeneration. Furthermore, the probability of observing regeneration depends strongly on the gap length; therefore data obtained by different investigators at different gap lengths cannot be compared on the same basis. On this basis, Yannas suggested the generation of a normalized database [151].

The need for biocompatibility of artificial nerve grafts can be described as following: (1) tubes should show timely vascularization, since early angiogenesis supports peripheral nerve regeneration. A time frame of 3-5 days seems to be adequate and is achieved by most of the materials. (2) Tubes must show a stable structure for extended nerve repair but should not cause mechanical irritation due to rigidity or collapse. Furthermore they should be easy to handle for clinical use. (3) Tubes should be biodegradable after successful nerve regeneration to avoid compression syndromes, but degradation products must not be carcinogenic or toxic or harmful to axonal growth. (4) Artificial or biological nerve tubes must be safe in terms of disease transmission, especial for tubes material derived from animal or human tissue.

Tumor formation associated with therapeutic clinical implants in humans are rare, despite the large numbers of medical implants used clinically over an extended period of time; nevertheless, cases of both human and veterinary implant-related tumors have been reported [152].

Most of regeneration studies face a common problem, namely a suboptimal animal model: Since experiments with rats are easy to realize, the rat sciatic nerve is the most commonly studied nerve in studies of induced regeneration in the peripheral nervous system combined with different immunohistochemical, histological, electrophysiological or neurological assessments. But rats have some physical limitations. Due to technical aspects the gap length separating the two nerve stumps inside the tube arbitrarily vary between 5 and 15 mm and it is very difficult to sufficiently bridge defects more than 2 cm. Since the probability of observing regeneration results depends strongly on the gap length, data obtained by different investigators at different gap lengths cannot be compared on the same basis. Moreover, most of these studies report about results “comparable to that of autografts”, especially with short gaps up to 10 mm and regardless of the tubes material. The “spontaneous” regeneration potential of rat nerves has to be kept in mind at the interpretation of regeneration results. Problems of peripheral nerve regeneration occur over long distances and these are the situations where Schwann cells, guiding structures and neurotrophic factors become more and more important. And so does the conduit material, which has to provide a stable scaffold with optimal tissue compatibility and permeability in order to maintain a suitable microenvironment inside the tubes for axonal regeneration. These nerve models with gaps more than 2 cm can only be realized in animals like dogs, rabbits or primates – facing logistic and ethical problems for studies with enough animals for statistical reliability and clinical long term observation for probably up to two years.

For clinical use, there is a need of studies with plenty of patients. So far only results of case reports or small collectives have been published. To assess effectiveness of FDA approved nerve tubes, large organized multicenter studies are required—which is difficult already due to internationally varying costs, starting from 500 Euros up to 2,000 Euros.

So far, no case has been published using autologous human Schwann cells in combination with a nerve tube – apparently due to technical, logistic and ethical considerations.

Once we have developed an “artificial nerve”, the question will be: “How can we accelerate peripheral nerve regeneration?” in order to prevent changes at the motor end plate and to grant a successful motor-target reinnervation within a reasonable time frame, since we know that irreversible changes at the target organs (e.g., muscle atrophy and fibrosis) can inhibit reinnervation. Another question is addressed to the specific axonal pathfinding of motor and sensory fibres inside the tube, so that a well regulated reinnervation is granted. Following injury of the peripheral nervous system such as nerve injury or amputation, the somatosensory cortex that responded to the deafferented body parts becomes responsive to neighboring body parts. Similarly, there is expansion of the motor representation of the stump area following amputation. Reorganization of the sensory and motor systems following peripheral injury occurs in multiple levels including the spinal cord, brainstem, thalamus and cortex. With successful nerve regeneration, these changes have to be reversible for a normal function.

In our opinion, lots of promising materials (acellular muscle, polymers and collagen) for nerve tubes are available, which leave space for necessary modifications (substructure, permeability, drug release, degradation) - but to really prove their potential, studies in large animal models with long nerve gaps are required.
